# Effectiveness and safety of nivolumab and ipilimumab in older adults with renal cell carcinoma: findings from a multicenter observational study in Poland

**DOI:** 10.3389/fonc.2025.1617743

**Published:** 2025-08-19

**Authors:** Artur Drobniak, Łukasz Stokłosa, Aleksandra Grela-Wojewoda, Jacek Calik, Natalia Versuti Viegas, Jolanta Dobrzańska, Agnieszka Roman, Marek Szwiec, Anna Bidas, Daria Tusień-Małecka, Angelika Gawlik-Urban, Mirosława Puskulluoglu, Renata Pacholczak-Madej

**Affiliations:** ^1^ Department of Chemotherapy, The District Hospital, Sucha Beskidzka, Poland; ^2^ Department of Chemotherapy, The Specialistic Hospital, Nowy Targ, Poland; ^3^ Department of Clinical Oncology, Maria Sklodowska-Curie National Research Institute of Oncology, Krakow, Poland; ^4^ Department of Clinical Oncology, University Hospital in Wroclaw, Wrocław, Poland; ^5^ Department of Oncology, Jagiellonian University Medical College, Krakow, Poland; ^6^ Oncology Clinical Department, The University Hospital, Krakow, Poland; ^7^ Department of Clinical Oncology, Ludwik Rydygier Hospital, Krakow, Poland; ^8^ Department of Surgery and Oncology, University of Zielona Góra, Zielona Góra, Poland; ^9^ Department of Clinical Oncology, Holy Cross Cancer Center, Kielce, Poland; ^10^ Clinical and Experimental Oncology Clinic, Institute of Oncology, Karol Marcinkowski Medical University, Poznań, Poland; ^11^ Clinical Oncology Department with Chemotherapy Subunit, Provincial Hospital Sain Luke, Tarnów, Poland; ^12^ Faculty of Health Protection, Tarnów University, Tarnów, Poland; ^13^ Department of Gynecological Oncology, Maria Sklodowska-Curie National Research Institute of Oncology, Krakow, Poland; ^14^ Department of Anatomy, Jagiellonian University, Medical College, Krakow, Poland

**Keywords:** renal cell carcinoma, nivolumab and ipilimumab, immune checkpoint inhibitors, elderly, treatment outcome

## Abstract

**Background:**

Nivolumab and ipilimumab (nivo+ipi) are recommended for treating metastatic clear cell renal cell carcinoma (mRCC), though their safety and efficacy in older adults remain uncertain. This study examines the outcomes of this regimen in Polish patients aged ≥65 years.

**Methods:**

In this multicenter observational study, 138 patients with mRCC who received nivo+ipi between May 2022 and October 2024 were analyzed. Key outcomes included objective response rates (ORR), disease control rate (DCR), and progression-free survival (PFS) with comparisons between patients aged <65 and ≥65 years. Safety was assessed based on the incidence and severity of immune-related adverse events (irAEs). Survival outcomes were analyzed using Kaplan-Meier methods and Cox proportional hazards models, adjusting for potential confounders. A significance level of p < 0.05 was applied.

**Results:**

After a median follow-up of 13 months, the median PFS for the entire cohort was 15.7 months (95% confidence interval [CI]: 10.2–20.8); in patients <65 years, it was 11.3 months, while in those ≥65 years, it was 23 months. Patients ≥65 years had a 40% lower risk of progression than younger patients (hazard ratio 0.6, 95% CI: 0.3–0.9, p=0.03). Patients aged ≥65 years exhibited a higher ORR (46.2% vs. 26%) and DCR (73.8% vs. 63%, p=0.02 for both). The overall incidence of irAEs was comparable between age groups; however, older patients experienced a higher frequency of very severe irAEs (1 vs. 6, p=0.06).

**Conclusions:**

This study demonstrates that nivo+ipi are effective across age groups, with older patients achieving comparable or even superior outcomes with acceptable irAEs rates.

## 1 Introduction

Immune checkpoint inhibitors (ICIs) play a well-established role in the treatment of clear cell renal cell carcinoma (RCC) in both adjuvant and palliative settings. Specifically, a combination of nivolumab and ipilimumab [an anti-programmed cell death receptor-1 (PD-1) and anti-cytotoxic T lymphocyte-associated antigen 4 (CTLA-4) antibodies] is recommended by international guidelines for intermediate- and poor-risk patients (1–3) according to the International Metastatic RCC Database Consortium (IMDC) criteria (4) (web calculator available at https://www.imdconline.com).

Although the median age at RCC diagnosis is 65 years (5), older patients are commonly underrepresented in clinical trials. Recent global estimates underscore a rising incidence in urinary malignancies, including kidney cancer, with aging identified as a major contributing factor to the projected burden by 2046 (6). There are theoretical concerns that the incidence of adverse events may be higher in older patients due to co-morbidities, use of other medications, functional impairments (decreased muscle mass, increased fat distribution), or reduced functional reserve (7). Additionally, immunosenescence, defined as the waning function of the immune system with advancing age, can theoretically impair the efficacy of ICIs (7). In the Checkmate 214 trial (8), which compared first-line treatment with nivolumab and ipilimumab and standard-of-care sunitinib (a vascular endothelial growth factor receptor tyrosine kinase inhibitor) in metastatic RCC (mRCC) across all risk groups, the median age of enrolled individuals who received combined immunotherapy was 62 (range: 26-85). However, patients ≥65 years old demonstrated poorer outcomes. Specifically, in terms of objective response rate (ORR) and overall survival (OS), this subgroup did not derive greater benefit from nivolumab and ipilimumab compared to sunitinib, as reported in the **Supplementary Appendix** of the trial. Conversely, the Checkmate 025 trial (9), which evaluated nivolumab versus everolimus (mammalian target of rapamycin inhibitor) in mRCC patients previously treated with one or two antiangiogenic therapies, showed a more pronounced progression-free survival (PFS) benefit of nivolumab in patients aged 65–75 years but not ≥75 years. Furthermore, real-world studies from Japan and Italy did not reveal worse outcomes or higher incidences of toxicities in older adults (10–12). However, due to variable pharmacokinetics, ICIs may have different clinical outcomes depending on geographic regions. Additionally, combined immunotherapy is associated with a risk of serious immune-related adverse events (irAEs) in 46% of patients (8) compared to 19% in nivolumab monotherapy (9), regardless of patients’ age.

This raises concerns regarding the utility and safety of this regimen in older adults. Therefore, we aimed to evaluate the efficacy and toxicity of the nivolumab and ipilimumab regimen in older individuals and compare the obtained results between patients ≥65 and those <65 years old.

## 2 Materials and methods

### 2.1 Patients

This observational study included 138 patients with clear cell mRCC (with or without sarcomatoid component) who received first-line combined immunotherapy within a national drug program of the Polish Ministry of Health (eligibility criteria for reimbursement are provided in the **Supplementary Materials**) (13). Eligible patients were treated with at least one cycle of nivolumab with ipilimumab with treatment initiation between May 1, 2022, and October 19, 2024, across nine oncology centers in Poland.

### 2.2 Ethical approval

The study protocol was approved by the Bioethics Committee of Jagiellonian University Medical College (approval number 118.0043.1.115.2024, dated April 19, 2024), and all patients provided institutional, informed consent before initiating nivolumab and ipilimumab treatment. We followed the European Society for Medical Oncology (ESMO) Guidance for Reporting Oncology Real-World Evidence (GROW) criteria to describe the obtained results (14), and detailed examples of their implementation are provided in **Supplementary Table S1** in the **Supplementary Materials**.

### 2.3 Data collection

The physicians collected data manually based on patients’ medical records, and the data cut-off was set on February 15, 2025. The research team reviewed the extracted data to ensure its completeness and accuracy. Missing data were managed using a complete-case analysis approach, where only patients with available data for relevant variables were included in the final analysis. Data regarding the patients’ baseline characteristics were recorded retrospectively with a prospective evaluation of the treatment course, response to therapy, and adverse events.

### 2.4 Study objectives

The primary objective was to assess the efficacy of the nivolumab and ipilimumab regimen and compare outcomes between patients aged ≥65 and <65 years old. Primary endpoints were the ORR and the disease control rate (DCR). The secondary endpoints included PFS, time to treatment failure (TTF) and OS. The secondary objective was to evaluate the safety profile in this patient cohort and compare outcomes between age subgroups.

### 2.5 Interventions

The treatment protocol followed European Union product guidelines (15, 16). Patients underwent an initial four-cycle induction phase with ipilimumab (1 mg/kg) and nivolumab (3 mg/kg) administered intravenously every three weeks. Following induction, patients continued with maintenance nivolumab at 480 mg every four weeks until progressive disease (PD), unacceptable toxicity or withdrawal of consent.

### 2.6 Outcome measures

The definitions of comorbidities documented as baseline characteristics are available in the **Supplementary Materials**.

ORR was defined as complete (CR) or partial response (PR) and the DCR as CR, PR or stable disease (SD) according to Response Evaluation Criteria in Solid Tumours (RECIST) 1.1 guidelines (17). OS was defined as the duration from the start of therapy to death; PFS was measured from the beginning of therapy to documented PD on a computed tomography (CT) scan or death; TTF was defined as the interval between treatment initiation and permanent discontinuation due to PD, treatment-related toxicity, patient death or withdrawal of consent. CT scans of the chest, abdomen and pelvis were conducted every 12 weeks or earlier if clinically indicated and underwent local assessment as per national drug program requirements (13).

Safety was assessed by recording irAEs from health records, categorized and graded according to the Common Terminology Criteria for Adverse Events (CTCAE) v.5.0 (18). IrAEs were classified into endocrine, hepatic, pulmonary, general (e.g., fatigue, infusion reactions, fever, decreased appetite), cutaneous, gastrointestinal (diarrhea/colitis), rheumatologic and hematologic groups.

Laboratory assessments were performed at local laboratories before treatment initiation to categorize patients into IMDC risk groups. Additionally, we calculated the neutrophil-to-lymphocyte ratio (NLR), platelet-to-lymphocyte ratio (PLR) and lymphocyte-to-monocyte ratio (LMR) by dividing the corresponding cell counts.

### 2.7 Bias and confounding

This study is subject to potential biases due to the retrospective nature of some data collection. To reduce selection bias, we included all eligible patients treated within the national drug program during the study period, with uniform, strict criteria across Poland, ensuring a representative sample. To minimize information bias, data were manually verified and cross-checked with treating physicians. Differences between centers were addressed by adhering to ESMO guidelines in the irAEs management (19) and using standardized RECIST v1.1 (17) criteria for tumor response assessment.

### 2.8 Follow-up

Patients were monitored during each treatment cycle or more frequently if clinically indicated. Follow-up for survival and adverse events continued until the data cut-off on February 15, 2025.

### 2.9 Statistical analysis

Statistical analyses were conducted using PS Imago Pro 9 (SPSS). Categorical variables were compared using Fisher’s exact test, chi-square test or proportion test to assess differences between the two age groups (<65 and ≥65 years). Continuous variables showed a non-normal distribution according to the Shapiro-Wilk test and were analyzed using the Mann-Whitney U test. PFS, TTF and OS were estimated using Kaplan-Meier methods, while Cox proportional hazards models and log-rank tests identified survival differences between the two age groups. Variables with p-value <0.05 in baseline comparisons were acknowledged as potential confounders and were included in multivariate Cox regression models. A p-value <0.05 was considered statistically significant.

## 3 Results

### 3.1 Patients’ characteristics

**Table 1** presents the baseline characteristics of patients, highlighting differences between those aged <65 years and ≥65 years. Older patients showed higher rates of comorbidities, including hypertension, hypercholesterolemia, hypothyroidism and a history of other malignancies. Additionally, these patients had poorer overall clinical status, as indicated by a lower Eastern Cooperative Oncology Group (ECOG) performance status and a Karnofsky Score <80%. The above factors were considered potential confounders. Apart from these differences, the two groups were well-balanced in other characteristics. The most common metastatic sites were the lungs, non-regional lymph nodes, bones and liver. A majority of patients underwent nephrectomy (83%) and were classified as intermediate risk according to IMDC criteria (74%). Laboratory parameters were also comparable between the two cohorts.

**Table 1:** Baseline patients’ characteristics.

				All patients n=138	<65 years n=73 (52.9%)	≥65 years n=65 (47.1%)	p-value
Demographics							
Age				64 (56-72)	57 (51-62)	72 (68-75)	0.001*
Males, n (%)				100 (72.5)	54 (74.0)	46 (70.8)	0.7
Females, n (%)				38 (27.5)	19 (26.0)	19 (29.2)	
Body mass index, kg/m^2^				26.8 (23.5-32.3)	26.8 (23.4-31.5)	26.9 (24.1-33.4)	0.5
Comorbidities							
Hypertension, n (%)				70 (50.7)	30 (41.1)	40 (61.5)	0.02*
Ischemic heart disease, n (%)				12 (8.7)	4 (5.5)	8 (12.3)	0.2
Heart failure, n (%)				6 (4.3)	4 (5.5)	2 (3.1)	0.5
Atrial fibrillation, n (%)				5 (3.6)	2 (2.7)	3 (4.6)	0.6
Hypercholesterolemia, n (%)				20 (14.5)	5 (6.8)	15 (23.1)	0.006*
Hypothyroidism, n (%)				15 (10.9)	2 (2.7)	13 (20.0)	0.001*
Diabetes mellitus type 2, n (%)				18 (13.0)	7 (9.6)	11 (16.9)	0.2
Venous thromboembolism, n (%)				9 (6.5)	6 (8.2)	3 (4.6)	0.4
Renal insufficiency, n (%)				31 (22.5)	13 (17.8)	18 (27.7)	0.2
Other malignancies, n (%)				4 (2.9)	0 (0)	4 (6.2)	0.03*
Baseline characteristics after radical treatment							
T stage after nephrectomy according to AJCC 8^th^ edition, n (%)	T1			13 (9.4)	5 (6.8)	8 (12.3)	0.4
	T2			12 (8.7)	6 (8.2)	6 (9.2)
	T3			78 (56.5)	47 (64.4)	31 (47.7)
	T4			6 (4.3)	4 (5.5)	2 (3.1)
	No data			29 (21.0)	11 (15.1)	18 (27.7)
Histologic subtype, n (%)	ccRCC			112 (81.2)	62 (84.9)	50 (76.9)	0.8
	ccRCC with sarcomatous components			19 (13.8)	11 (15.1)	8 (12.3)
	No data			7 (5.1)	0 (0)	7 (10.8)
Characteristics at the time of treatment initiation							
Performance status, n (%)		0		18 (13.0)	14 (19.2)	4 (6.1)	0.001*
		1		103 (74.6)	56 (76.7)	47 (72.3)	
		2		17 (12.3)	3 (4.1)	14 (21.5)	
Nephrectomy, n (%)		Yes		115 (83.3)	60 (82.2)	55 (84.6)	0.7
		No		23 (16.7)	13 (17.8)	10 (15.4)	
Time from nephrectomy to treatment initiation (months)				3.1 (1.7-7.4)	2.6 (1.7-5.9)	3.3 (1.7-10.0)	0.2
Primary metastatic, n (%)				81 (58.7)	47 (64.4)	34 (52.3)	0.2
Number of sites with target/non-target lesions n (%)	≤ 2			66 (47.8)	32 (43.8)	34 (52.3)	0.3
	> 2			72 (52.2)	41 (56.2)	31 (47.7)	
Site of metastasis at the baseline CT scan, n (%)	Nonregional Lymph nodes			60 (43.5)	28 (38.4)	32 (49.2)	0.2
	Adrenal glands			28 (20.3)	19 (26.0)	9 (13.8)	0.08
	Liver			31 (22.5)	13 (17.8)	18 (27.7)	0.2
	Central nervous system			8 (5.8)	5 (6.8)	3 (4.6)	0.6
	Lungs			91 (65.9)	47 (64.4)	44 (67.7)	0.7
	Bones			37 (26.8)	21 (28.8)	16 (24.6)	0.6
IMDC risk group, n (%)	Intermediate			102 (73.9)	55 (75.3)	47 (72.3)	0.7
	Poor			36 (26.1)	18 (24.7)	18 (27.7)
Number of risk factors, n (%)	1			52 (37.7)	28 (38.4)	24 (36.9)	0.9
	2			51 (37.0)	28 (38.4)	23 (35.4)
	3			26 (18.8)	12 (16.4)	14 (21.5)
	4			9 (6.5)	5 (6.8)	4 (6.2)
No of patients with risk categories, n (%)	Time from the diagnosis to treatment onset <1 year			118 (85.5)	65 (89.0)	53 (81.5)	0.2
	Karnofsky Score <80%			25 (18.1)	6 (8.2)	19 (29.2)	0.001*
	Hemoglobin level <unl			80 (58.0)	43 (58.9)	37 (56.9)	0.8
	Corrected calcium >unl			11 (8.0)	7 (9.6)	4 (6.2)	0.5
	Neutrophils >unl			14 (10.1)	10 (13.7)	4 (6.2)	0.1
	Platelets >unl			27 (19.6)	16 (21.9)	11 (16.9)	0.5
Selected laboratory parameters	NLR			2.8 (1.9-3.7)	2.8 (1.9-3.5)	2.8 (1.9-4)	0.9
	PLR			168.9 (119.4- 240.3)	172.6 (127.6- 234.7)	168.9 (113.4-262.6)	0.6
	LMR			2.8 (1.9-3.7)	2.8 (1.9-3.7)	2.8 (2-3.5)	1
	Eosinophils (10^3^/ul)			0.2 (0.1-0.2)	0.2 (0.1-0.2)	0.2 (0.1-0.2)	0.7
	Monocytes (10^3^/ul)			0.6 (0.5-0.8)	0.6 (0.5-0.8)	0.7 (0.5-0.8)	0.4

Categorical variables are presented as numbers (percentages), and continuous variables are presented as medians and interquartile ranges.

Values with statistical significance are marked as *.

AJCC, American Joint Committee on Cancer; ccRCC, clear cell renal cell carcinoma; CT, computed tomography; IMDC, International Metastatic Renal Cell Carcinoma Database Consortium; LMR, lymphocyte-to-monocyte ratio; NLR, neutrophil-to-lymphocyte ratio; n, number; PLR, platelet-to-lymphocyte ratio; T, tumor; unl, upper normal limit.

### 3.2 Treatment efficacy

Patients were followed for a median of 13 months (interquartile ranges [IQR]: 7.4–22.4). The median treatment duration was 5.9 months (IQR: 2.1–11.8) and patients received a median of 6 treatment cycles (IQR: 4–12). No significant differences were observed between the two age cohorts in these parameters (p=0.3, p=0.3, and p=0.9, for a follow-up period, treatment duration and number of cycles, respectively).

Two-thirds of the enrolled patients (n=94, 68.1%) achieved disease control after the nivolumab + ipilimumab treatment regimen. As shown in **Table 2**, patients aged ≥65 years exhibited a higher ORR (46.2% vs. 26%) and DCR (73.8% vs. 63%, p=0.02 for both) and they were more likely to demonstrate a PR on CT scans (40% vs. 23.3%, p=0.03). PD as the best overall response was significantly more frequent in younger patients (27.4% vs. 13.8%, p=0.05). At the data cutoff, 55 events of PD were recorded, with 36 events in patients <65 years and 19 events in patients ≥65 years (49.3% vs. 29.2%, p=0.02).

**Table 2:** The treatment outcome with nivolumab and ipilimumab in all patients and two age cohorts.

	All patients n=138	<65 years n=73 (52.9%)	≥65 years n=65 (47.1%)	p-value					
Overall response rate, n (%)	49 (35.5)	19 (26.0)	30 (46.2)	0.02*
Disease control rate, n (%)	94 (68.1)	46 (63.0)	48 (73.8)	0.02*
Best overall response									
Complete remission, n (%)	6 (4.3)	2 (2.7)	4 (6.2)	0.3
Partial response, n (%)	43 (31.2)	17 (23.3)	26 (40.0)	0.03*
Stable disease, n (%)	45 (32.6)	27 (37.0)	18 (27.7)	0.2
Progressive disease, n (%)	29 (21.0)	20 (27.4)	9 (13.8)	0.05*
N/A, n (%)	15 (10.9)	7 (9.6)	8 (12.3)	0.6

Categorical variables are presented as numbers (percentages).

n, number; N/A, not accessed.

Values with statistical significance are marked as *.

**Figure 1:**
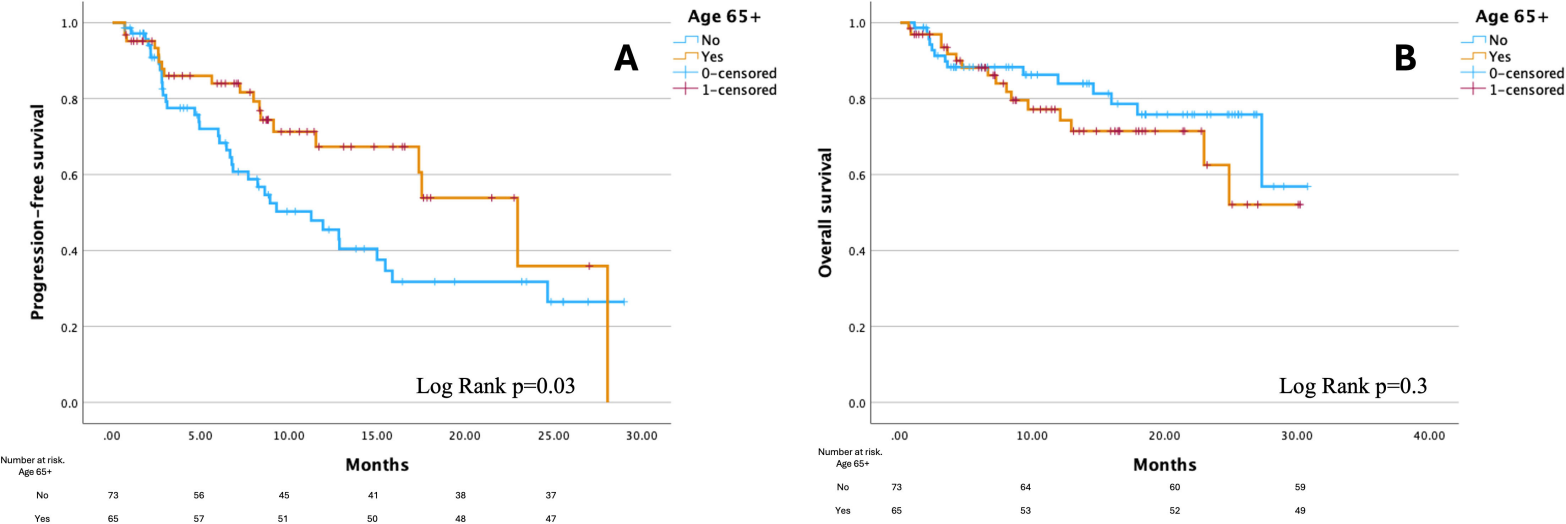
Kaplan-Meier curves for progression-free survival **(A)** and overall survival **(B)** by age group. Figure created using PS Imago Pro 9 (SPSS).

The median PFS for the entire cohort was 15.7 months (95% confidence interval [CI]: 10.2–20.8); for patients <65 years, it was 11.3 months (95% CI: 6.7–15.8) and for patients ≥65 years, it was 23 months (95% CI: 15.8–30.2), (**Figure 1A**). Patients ≥65 years had a 40% lower risk of disease progression than younger patients (hazard ratio [HR] 0.6, 95% CI: 0.3–0.9, p=0.03) and this value remained significant also in the multivariate regression model with p=0.02 (details in **Supplementary Table S2** in the **Supplementary Materials**) after adjustment for potential confounders identified in baseline differences between two cohorts (hypertension, hypothyroidism, hypercholesterolemia, other malignancies and performance status). The median TTF was 11.9 months (95% CI: 6.7-17.2) for the entire cohort; for patients <65 years, it was 8.9 months (95% CI: 5.1-12.8) and for patients ≥65 years, it was 17.5 months (95% CI: 11-24.1) without differences between two subgroups (log-rank p=0.2).

Median OS was not reached (NR) for the entire cohort or either age group. No significant difference in survival was observed between the two age cohorts (log-rank p=0.3, **Figure 1B**) and this remained consistent after adjustment for confounders in the multivariate regression model (details in **Supplementary Table S3** of the **Supplementary Materials**). The 6-month survival rate for the entire cohort was 89%, while the 12-month survival rate was 84%. Among patients <65 years, the 6-month survival rate was 89% and the 12-month survival rate was 86%. In patients ≥65 years, the corresponding survival rates were 89.2% at 6 months and 81.5% at 12 months. At the data cutoff, there were 30 recorded deaths: 14 in patients <65 years and 16 in patients ≥65 years (19.2% vs. 24.6%, p=0.5).

### 3.3 Safety

In total, 77 patients experienced irAEs (42 patients <65 years and 35 patients ≥65 years, p=0.8), accounting for 123 events (63 events in patients <65 years and 60 in patients ≥65 years, p=0.9). A single episode occurred in 47 patients, two in 17, three in 10 and four in 3.

The majority of irAEs were mild (grade, G1), recorded in 57 cases (30 in patients <65 years and 27 in those, p=0.8). Moderate irAEs (G2) were documented in 36 cases (20 vs. 16, p=0.6). Severe events (G3) were seen in 23 cases (11 vs. 12, p=1.00), while very severe irAEs (G4) were noted in 7 cases (1 vs. 6, p=0.06).

**Figure 2:**
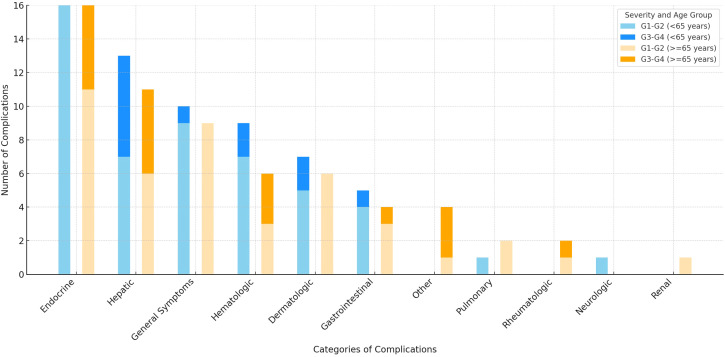
Number of immune-related adverse events by severity and age group graded according to Common Terminology Criteria for Adverse Events v.5.0. Figure created using Microsoft Excel, version 16.99 (Microsoft Corporation, Redmond, WA, USA). Abbreviations: G- grade.

**Figure 2** illustrates the distribution of irAEs across adverse events categories, grouped by age (<65 years and ≥65 years) and severity (G1-G2 and G3-G4). Analysis of event rates across irAEs categories showed a statistically significant difference between age groups in the endocrine category (p=0.04). No statistically significant differences were found in other categories (details in **Supplementary Table S4** in the **Supplementary Materials**).

In the Cox regression model, the occurrence of irAEs did not significantly impact PFS in the entire cohort (p=0.5), in patients <65 years (p=0.4) or in those ≥65 years (p=0.9). A similar trend was observed for OS (p=0.8 for the entire cohort, p=0.9 for patients <65 years and p=0.8 for those ≥65 years).

Systemic steroids were administered to 29 patients (21%), including 17 patients <65 years (23.3%) and 12 patients ≥65 years (18.5%), with p=0.5. The median steroid dose was 1 mg/kg of prednisone/equivalent (IQR: 0.75–2 mg/kg). Two patients (1.4%) required treatment with mycophenolate mofetil. No other immunosuppressive treatment was administered in this cohort.

### 3.4 Treatment discontinuation and subsequent therapy in the entire cohort

Sixty-eight patients (49.3%) continued treatment at the data cut-off, while 70 patients (50.7%) had treatment withdrawal, primarily due to PD/death (n=55, 78.6%) or toxicity (n=15, 21.4%). Of those who discontinued, 29 patients (41.4%) began a subsequent line of therapy with cabozantinib (n=27) or axitinib (n=2) and six patients (8.6%) are under active surveillance and expected to start the next treatment line in case of PD. A total of 35 patients (50%) were ineligible for further treatment, with 30 of them having already passed away.

## 4 Discussion

In this analysis, we observed significant differences in treatment outcomes and safety profiles between younger and older patients with mRCC undergoing nivolumab and ipilimumab treatment. Older patients, despite having higher rates of comorbidities and poorer baseline performance status, achieved comparable or even favorable treatment outcomes relative to their younger counterparts. Specifically, patients aged ≥65 demonstrated a higher ORR and DCR, alongside a longer PFS, with age being identified as an independent prognostic factor for disease progression. Safety outcomes revealed that the frequency of irAEs was similar across age groups, with most of them being mild and moderate (G1-G2). Older patients experienced more severe adverse events and higher incidences of cardiac events such as myocarditis and pericarditis.

Traditionally, the term “elderly” refers to individuals aged 65 and older (20, 21) but with the aging population, many studies have redefined “older patients” as those over 70–75 years (10, 11). In the pivotal Checkmate 214 trial (8), patients were categorized into three age groups (<65 years, ≥65 and <75 years and ≥75 years), where those over 65 showed no significant ORR and OS benefit from nivolumab and ipilimumab. Additionally, in our recent report (22), we found that age ≥65 was linked to a significantly reduced risk of disease progression (HR=0.5, 95% CI: 0.3-0.8, p=0.01). Accordingly, we chose this landmark for our analysis and aimed to further explore this observation in the current study.

Our findings appear to contrast with the results of the pivotal CheckMate 214 trial (8). Several factors may help explain this difference. First, real-world populations may differ from clinical trial cohorts in terms of baseline characteristics and comorbidities. In our study, treatment decisions were made in routine practice and may have favored patients with preserved organ function and better performance status. Second, improvements in the recognition and management of irAEs since the time of the CheckMate 214 (8) trial may have contributed to better outcomes in real-world settings.

In a network meta-analysis (23), most first-line treatments had less favorable outcomes in older patients, yet the combination of nivolumab + ipilimumab followed by second-line cabozantinib showed the greatest efficacy in this population. Additionally, in the large Italian multicenter study, patients who were older than 70 years had comparable OS to younger patients (12), like in our study. However, OS is influenced by multiple factors, including additional lines of therapy and deaths from non-cancer-related comorbidities. Consequently, PFS may serve as a more accurate measure of the direct impact of nivolumab and ipilimumab in this population.

In an exploratory analysis of patients with mRCC, melanoma and non-small cell lung cancer treated with nivolumab in clinical trials, the incidence of irAEs was similar in patients aged <65 and ≥65; however, G3–5 adverse events were more frequent in patients over 70 (58.4% vs. 71.7%) (24) what stays in line with our findings and in our previous report (25). In a cohort of 103 melanoma patients aged ≥80 who received either single-agent ICIs or nivolumab + ipilimumab, the adverse event rate was consistent with phase III data across all age groups (26). In other reports of melanoma patients, survival and toxicity in older patients treated with ICIs were similar to younger patients (27, 28). While growing evidence suggests that irAEs occurrence may correlate with improved outcomes (29–32), Johns et al. (33) did not find this relationship in patients aged ≥70 (similar to our findings), contrasting with Schulz et al. (34) who did observe it in patients with genitourinary cancers, though only 10 of these patients received combination immunotherapy. We may hypothesize that improved outcomes in older adult patients are related to higher rates of irAEs with predictive value, as we previously reported (30).

As the immune system ages, it undergoes metabolic and structural changes, collectively known as immunosenescence. Key alterations include thymic atrophy, shifts in T and B cell ratios, reduced hematopoietic stem cell function, mitochondrial dysfunction and increased inflammation. These changes weaken immune efficiency and reduce the body’s ability to respond to new antigens, including tumors, leading to fewer tumor-infiltrating immune cells and potentially reducing the effectiveness of some immunotherapies (35). However, chronic low-grade inflammation, a feature of immunosenescence, may paradoxically enhance immunotherapy response in older adults by promoting inflammatory factors, like interleukin (IL)-1, IL-6 and IL-8, through the senescence-associated secretory phenotype (SASP) (36–38). SASP components in the tumor microenvironment have been linked to tumor progression and drug resistance, yet may also limit aggressive tumor growth, as seen in older patients with bronchial cancer and elderly mouse cancer models, where slower tumor growth and fewer metastases were observed (39, 40). In terms of immune checkpoint dynamics, certain checkpoints like CTLA-4 appear stable with age, while slight changes in PD-1 and increases in PD-L1 and PD-L2 expression have been observed, suggesting that therapies targeting these checkpoints may remain effective in older patients (7, 41, 42). These observations might explain why our study found better treatment outcomes in older patients, as their unique immune landscape and altered inflammatory profile could improve responsiveness to ICIs. However, no differences were observed in inflammatory markers derived from the complete blood count, including the NLR, PLR, LMR, eosinophil counts or monocyte counts. Currently, in the novel Meet-URO Score, NLR has been added, providing higher prognostic accuracy (web calculator available at: https://proviso.shinyapps.io/Meet-URO15_score/) (43). Further studies are needed to confirm these findings.

## 5 Study limitations

The primary limitation of this study is the relatively small sample size, which may reduce statistical power and impact the robustness of our findings. Additionally, the retrospective data collection for certain variables may introduce recall or selection bias. While our findings contribute important information on PFS and OS, the follow-up period may not be long enough to capture delayed effects or longer-term outcomes associated with the nivolumab and ipilimumab regimen. We acknowledge that the retrospective nature of the study and variability in imaging schedules may have influenced the accuracy of PFS estimates and the relatively short follow-up period limits the maturity of OS data. The absence of a standardized comorbidity index (e.g., Charlson Comorbidity Index) limits the granularity of our assessment, as comorbidities were retrospectively extracted from medical records without formal geriatric evaluation. Assessments of safety, laboratory and imaging were conducted locally, which may introduce reporting bias and variability in assessments. Variations in the management of irAEs could influence outcomes, as local practices may differ in steroid administration or other interventions for irAEs. The study’s restriction to a Polish cohort and the necessity to strictly adhere to reimbursement criteria may also limit the generalizability of results to mRCC populations in other regions with differing genetic backgrounds, environmental exposures and healthcare practices. Furthermore, despite the use of multivariate analyses, unmeasured confounding factors could still influence the observed outcomes. Propensity score matching (PSM) in statistical analysis was not applied due to the relatively small sample size, which would have led to a significant reduction in the number of analyzed patients and decreased statistical power. Instead, multivariate Cox regression models were used to adjust for potential confounders while preserving the full dataset. Additionally, as this is a real-world study, applying PSM could introduce selection bias by excluding patients without a matched counterpart, limiting the generalizability of the findings.

## 6 Conclusions

This study demonstrates that nivolumab and ipilimumab are effective across age groups, with older patients (≥65 years) achieving comparable or even better ORR, DCR and PFS than younger patients despite higher comorbidity rates. However, older patients were more susceptible to severe irAEs, highlighting the need for close monitoring and personalized management in this group. These findings support immunotherapy as a viable option for older patients and that chronological age should not be a direct contraindication for such treatment. As a pilot multicenter study, our findings offer preliminary insight into age-specific treatment outcomes and warrant confirmation in larger, prospective cohorts.

## Data Availability

The raw data supporting the conclusions of this article will be made available by the authors, without undue reservation.
