# Supplementary material for: Effectiveness and safety of nivolumab and ipilimumab in older adults with renal cell carcinoma: findings from a multicenter observational study in Poland
Source: Front Oncol. 2025 Aug 19;15:1617743. doi: 10.3389/fonc.2025.1617743 (PMC12401697; doi:10.3389/fonc.2025.1617743)
Supplement: Supplementary file 1 [file DataSheet1.docx]

Supplementary Material

**1 Reimbursement criteria for nivolumab and ipilimumab combined immunotherapy according to the national drug program of the Polish Ministry of Health in patients with renal cell carcinoma in Poland** [1].

1.1. General eligibility criteria

- Histologically confirmed diagnosis:
  - Renal cell carcinoma with clear cell or sarcomatoid component.
- Advanced tumor stage: no possibility for radical local treatment.
- No prior pharmacological treatment.
- Performance status: Karnofsky score 70-100.
- Age: 18 years or older.
- Primary tumor removal: previous tumor resection or documented multidisciplinary decision against nephrectomy.
- Measurable lesions: objective assessment possible per Response Evaluation Criteria in Solid Tumours (RECIST) [2].
- No active central nervous system (CNS) metastases: previous resection or stereotactic radiotherapy allowed if asymptomatic post-treatment.
- Concurrent active malignancies: treatment eligibility must consider prognosis related to coexisting malignancy.
- No significant comorbidities: no clinical conditions contraindicating therapy per the current summary of product characteristics (SmPC).
- Adequate organ function: based on blood laboratory results per current SmPC.
- Exclusion of pregnancy and breastfeeding: confirmed non-pregnant and not breastfeeding.
- Patient agreement: contraceptive use agreement per current SmPC of administered drugs.

1.2. Specific eligibility criteria for therapy

- Intermediate or poor prognosis according to the International Metastatic RCC Database Consortium (IMDC) scale.
- No prior treatment with immune checkpoint inhibitors, including adjuvant therapy.
- No active autoimmune diseases, except Sjögren's syndrome, vitiligo, type I diabetes, hypothyroidism (treated only with hormone supplementation), psoriasis not requiring systemic treatment.
- No systemic corticosteroids (exceeding 10 mg prednisone daily or equivalent) or immunosuppressive drugs within 14 days before starting treatment (inhaled corticosteroids are allowed).
- No history of hypersensitivity to monoclonal antibodies.

2. Treatment duration in the program

Treatment continues until the attending physician decides to exclude the patient from the program, according to exclusion criteria.

3. Exclusion Criteria from the Program

- Documented disease progression per current RECIST criteria [2] (confirmation of progression in another assessment after 4-8 weeks is possible).
- In the case of oligoprogression (progression in no more than 5 foci in no more than 3 organs, including the CNS), treatment continuation is allowed if radical local treatment (surgery, stereotactic radiotherapy) is applied to these foci, provided the patient benefits clinically and other lesions remain responsive or stable.
- Hypersensitivity to any active substance or excipient.
- Pregnancy or breastfeeding.
- Life-threatening toxicity or significant clinically relevant lower-grade toxicity that recurs despite appropriate management per current SmPC or oncology society guidelines.
- Persistent significant deterioration in general performance or quality of life preventing treatment continuation.
- Occurrence of diseases or conditions that, in the attending physician's opinion, preclude treatment.
- Non-cooperation or non-compliance with medical recommendations, particularly regarding periodic control examinations to assess treatment efficacy and safety.

Additionally, patients treated with active substances funded in the drug program through other financing methods, except clinical trials for first-line treatment, are eligible for the drug program, provided they met the eligibility criteria at treatment initiation.

1. Dosage

The method of administration and any temporary treatment interruptions should follow the current SmPC or accepted clinical practice. Dose reductions of the medications are permissible according to the current SmPC.

1. Qualification Tests

- Histopathological diagnosis of renal cell carcinoma;
- Complete blood count with differential;
- Serum creatinine level;
- Serum bilirubin level;
- Alanine aminotransferase activity;
- Aspartate aminotransferase activity;
- Lactate dehydrogenase activity;
- Thyroid-stimulating hormone (TSH) and thyroxine (fT4) levels;
- Corrected serum calcium level;
- Alkaline phosphatase activity;
- Blood glucose level;
- Other laboratory tests if clinically indicated;
- Pregnancy test for women of childbearing potential (if clinically indicated);
- Electrocardiogram;
- Blood pressure measurement;
- Computed tomography (CT) scan of the chest and abdomen;
- Chest X-ray – only if it allows measurement of lesions and assessment of treatment response;
- CT or magnetic resonance imaging (MRI) of the brain – for patients suspected of central nervous system metastases (i.e., with CNS symptoms or after prior local treatment of metastases);
- Other imaging studies if clinically indicated.

Initial imaging studies must enable later objective assessment of treatment response according to current RECIST criteria.

6. Monitoring Treatment Safety

- Complete blood count with differential;
- Serum creatinine level;
- Serum bilirubin level;
- Alanine aminotransferase activity;
- Aspartate aminotransferase activity;
- fT4 and TSH levels;
- Blood pressure measurement;
- Other tests if clinically indicated.

Tests are conducted every 3-6 weeks – during combination therapy with ipilimumab and nivolumab.

Safety monitoring tests can be performed more frequently if clinically indicated.

7. Monitoring Treatment Effectiveness

- CT or MRI of the relevant area;
- Chest X-ray – if no CT scan is performed;
- Other imaging studies if clinically indicated.

Tests are conducted:

- At least every 12 weeks (with possible delay up to 2 weeks if treatment interruptions occur);
- If treatment extends beyond 3 years, imaging tests can be performed at least every 6 months (with possible delay up to 4 weeks if treatment interruptions occur);
- At the time of program discontinuation, unless due to documented disease progression, and always if clinically indicated.

The conducted imaging studies must enable objective assessment of treatment response. Treatment response evaluation should follow current RECIST criteria [2].

8. Program Monitoring

- Collecting treatment monitoring data in medical records and presenting them on request to National Health Fund (NHF) inspectors;
- Updating data in the electronic drug program monitoring system available via an internet application provided by NHF, as per program description and at treatment termination;
- Reporting and billing information to NHF (in paper or electronic form) as per NHF requirements;

**2 Comorbidities** [3]

- Arterial hypertension- history of blood pressure > 140/90 mmHg or current antihypertensive treatment);
- Ischemic heart disease - symptoms of chest pain or myocardial infarction in the past;
- Heart failure - ejection fraction < 50%;
- Atrial fibrillation- diagnosed in the past during active antiarrhythmic treatment;
- Hypercholesterolemia - a total serum cholesterol > 5.2 mmol/L or ongoing antihypercholesterolemic therapy;
- Hypothyroidism - current use of hormone replacement therapy;
- Diabetes mellitus type 2- use of insulin or oral hypoglycemic agents, or fasting serum glucose > 7.0 mmol/L;
- Venous thromboembolism- previously diagnosed and managed with anticoagulation therapy;
- Renal insufficiency- glomerular filtration rate [GFR] lower than 90 but higher than 60 mL/min/1.73 m^2^;
- Other malignancies- reported in patients’ history, after radical treatment;

Table S1. Key elements of real-world evidence reporting based on European Society for Medical Oncology Guidance for Reporting Oncology Real-World Evidence (ESMO-GROW) guidelines with examples from the study methodology [4].

| **GROW Element** | **Description** | **Example from Methods Section** |
| --- | --- | --- |
| **Study Context** | Clearly define the setting, location, and time frame of the study. | “This observational study included 138 patients with clear cell mRCC (with or without sarcomatoid component) … with treatment imitation between May 1, 2022, and October 19, 2024, across nine oncology centers in Poland.” |
| **Study Population** | Provide detailed inclusion and exclusion criteria, and describe the representativeness of the population. | "This observational study included 138 patients with clear cell mRCC (with or without sarcomatoid component) who received first-line combined immunotherapy within a national drug program of the Polish Ministry of Health (eligibility criteria for reimbursement are provided in Supplementary Materials) [1]. Eligible patients were treated with at least one cycle of nivolumab with ipilimumab with treatment initiation between May 1, 2022, and October 19, 2024, across nine oncology centers in Poland.” |
| **Study Design** | State the design (e.g., retrospective, prospective) and adherence to guidelines. | " data regarding the patients’ baseline characteristics were recorded retrospectively with a prospective evaluation of the treatment course, response to therapy, and adverse events occurrence.” |
| **Ethical Considerations** | Detail ethical approvals and patient consent. | "The study protocol was approved by the Bioethics Committee of Jagiellonian University Medical College (approval number 118.0043.1.115.2024, dated April 19, 2024), and all patients provided institutional, informed consent before initiating nivolumab and ipilimumab treatment" |
| **Data Sources and Quality** | Specify data sources, data collection methods, and quality assurance processes. | " The physicians collected data manually based on patients’ medical records, and the data cut-off was set on February 15, 2025. The research team reviewed the extracted data to ensure its completeness and accuracy" |
| **Handling Missing Data** | Describe methods for addressing missing data and their impact. | " Missing data were managed using a complete-case analysis approach, where only patients with available data for relevant variables were included in the final analysis.” |
| **Study Objectives** | Define primary and secondary objectives and endpoints. | " The primary objective was to assess the efficacy of the nivolumab and ipilimumab regimen and compare outcomes between patients aged <65 and ≥65 years. Primary endpoints were the ORR and the disease control rate (DCR). The secondary endpoints included PFS, time to treatment failure (TTTF) and OS. The secondary objective was to evaluate the safety profile in this patient cohort and compare outcomes between age subgroups." |
| **Interventions** | Provide details of treatments and protocols followed. | " The treatment protocol followed European Union product guidelines. Patients underwent an initial four-cycle induction phase with ipilimumab (1 mg/kg) and nivolumab (3 mg/kg) administered intravenously every three weeks." |
| **Outcome Measures** | Describe how outcomes were defined, measured, and assessed. | "The definitions of comorbidities documented as baseline characteristics are available in the Supplementary Materials. OS was defined as the duration from the start of therapy to death, while PFS was measured from the beginning of therapy to documented disease progression on a computed tomography (CT) scan or death… Safety was assessed by recording irAEs from health records, categorized and graded according to the Common Terminology Criteria for Adverse Events (CTCAE) v.5.0… Laboratory assessments were performed at local laboratories before treatment initiation to categorize patients into IMDC risk groups …" |
| **Bias and Confounding** | Address potential biases and strategies to minimize them. | " This study is subject to potential biases due to the retrospective nature of some data collection. To reduce selection bias, we included all eligible patients treated within the national drug program during the study period, with uniform, strict criteria across Poland, ensuring a representative sample. To minimize information bias..." |
| **Safety Assessments** | Define how adverse events were classified and graded. | " Safety was assessed by recording irAEs from health records, categorized and graded according to the Common Terminology Criteria for Adverse Events (CTCAE) v.5.0 [5]. IrAEs were classified into endocrine, hepatic, pulmonary, general…." |
| **Follow-up Period** | State the duration and frequency of follow-up and assessments. | " Patients were monitored during each treatment cycle or more frequently if clinically indicated. Follow-up for survival and adverse events continued until the data cut-off on February 15, 2025.” |

Table S2. Univariate and multivariate Cox regression model for progression-free survival with factors identified as potential confounders in baseline characteristics.

|  | Hazard ratio | 95% CI | p-value |
| --- | --- | --- | --- |
| Univariate regression model | | | |
| Age≥65 years | 0.6 | 0.3-0.9 | 0.03* |
| Karnofsky Score <80% | 1.3 | 0.7-2.6 | 0.4 |
| Hypertension | 0.7 | 0.4-1.2 | 0.2 |
| Hypothyroidism | 0.7 | 0.3-1.8 | 0.5 |
| Hypercholesterolemia | 0.3 | 0.08-0.9 | 0.03* |
| Other malignancies | 0.7 | 0.2-3.1 | 0.7 |
| Multivariate regression model, p=0.03 | | | |
| Age≥65 years | 0.5 | 0.2-0.9 | 0.02* |
| Karnofsky Score <80% | 1.9 | 0.9-4.0 | 0.09 |
| Hypertension | 1 | 0.5-1.7 | 0.9 |
| Hypothyroidism | 1.1 | 0.4-3.1 | 0.8 |
| Hypercholesterolemia | 0.2 | 0.06-0.9 | 0.04* |
| Other malignancies | 3 | 0.6-15.4 | 0.2 |

Abbreviations: CI- Confidence interval
Values with statistical significance are marked as *.

Table S3. Univariate and multivariate Cox regression model for overall survival with factors identified as potential confounders in baseline characteristics.

|  | Hazard ratio | 95% CI | p-value |
| --- | --- | --- | --- |
| Univariate regression model | | | |
| Age≥65 years | 1.5 | 0.7-3 | 0.3 |
| Karnofsky Score <80% | 2.3 | 1.1-5 | 0.04* |
| Hypertension | 1 | 0.5-2.1 | 0.9 |
| Hypothyroidism | 1.5 | 0.5-4.3 | 0.5 |
| Hypercholesterolemia | 0.2 | 0.03-1.4 | 0.1 |
| Other malignancies | 0.05 | 0.2-15 | 0.4 |
| Multivariate regression model, p=0.07 | | | |
| Age≥65 years | 0.6 | 0.3-1.1 | 0.08 |
| Karnofsky Score <80% | 0.9 | 0.5- 1.6 | 0.7 |
| Hypertension | 1 | 0.6- 1.7 | 0.9 |
| Hypothyroidism | 1 | 0.4- 2.8 | 0.9 |
| Hypercholesterolemia | 0.2 | 0.06-0.9 | 0.04 |
| Other malignancies | 2.3 | 0.5-11.3 | 0.3 |

Abbreviations: CI- Confidence interval
Values with statistical significance are marked as *.

Table S4. Immune-related adverse event (irAEs) rates by complication type, grade according to the Common Terminology Criteria for Adverse Events (CTCAE) v.5.0 [5], age group, and p-values with percentages.

| **Adverse event category** | **<65  G1-G2 n=50** | **≥65**  **G1-G2**  **n=43** | **p-value G1-G2** | **<65**  **G3-G4 n=12** | **≥65 G3-G4**  **n=18** | **p-value G3-G4** |
| --- | --- | --- | --- | --- | --- | --- |
| Endocrine n=32, n(%) | 16 (32) | 11 (25.6) | 0.04* | 0 | 5 (62.5)^1^ | 0.33 |
| Hepatic n=24, n(%) | 7 (14) | 6 (14) | 1.0 | 6 (50) | 5 (62.5) | 1.0 |
| General Symptoms^2^ n=19, n(%) | 9 (18) | 9 (20.9) | 1.0 | 1 (8.3) | 0 | 1.0 |
| Hematologic n=15, n(%) | 7 (14) | 3 (7) | 0.33 | 2 (16.7) | 3 (37.5) | 0.46 |
| Dermatologic n=13, n(%) | 5 (10) | 6 (14) | 0.46 | 2 (16.7) | 0 | 1.0 |
| Gastrointestinal n=9, n(%) | 4 (8) | 3 (7) | 1.0 | 1 (8.3) | 1 (12.5) | 1.0 |
| Other^3^ n=4, n(%) | 0 | 1 (2.3) | 1.0 | 0 | 3 (37.5) | 1.0 |
| Pulmonary n=3, n(%) | 1 (2) | 2 (4.7) | 1.0 | 0 | 0 | 1.0 |
| Rheumatologic n=2, n(%) | 0 | 1 (2.3) | 1.0 | 0 | 1 (12.5) | 1.0 |
| Neurologic n=1, n(%) | 1 (2) | 0 | 1.0 | 0 | 0 | 1.0 |
| Renal n=1, n(%) | 0 | 1 (2.3) | 1.0 | 0 | 0 | 1.0 |

Abbreviations: G- grade, n- numer
Values with statistical significance are marked as *.
1- G3-G4 endocrine irAEs: hypophysitis (n=4) and adrenal insufficiency (n=1)

2- General Symptoms: fatigue (n=13), infusion reactions (n=2), fever (n=2), decreased appetite (n=2)

3- Other irAEs: myocarditis (n=3), pericarditis (n=1)

**References**

1. Obwieszczenie Ministra Zdrowia z dnia 18 grudnia 2024 r. w sprawie wykazu refundowanych leków, środków spożywczych specjalnego przeznaczenia żywieniowego oraz wyrobów medycznych na 1 stycznia 2025 r. - Ministerstwo Zdrowia - Portal Gov.pl. https://www.gov.pl/web/zdrowie/obwieszczenie-ministra-zdrowia-z-dnia-18-grudnia-2024-r-w-sprawie-wykazu-refundowanych-lekow-srodkow-spozywczych-specjalnego-przeznaczenia-zywieniowego-oraz-wyrobow-medycznych-na-1-stycznia-2025-r [Accessed December 26, 2024]

2. Somarouthu B, Lee SI, Urban T, Sadow CA, Harris GJ, Kambadakone A. Immune-related tumour response assessment criteria: a comprehensive review. Br J Radiol (2018) 91:736. doi: 10.1259/BJR.20170457

3. Podręcznik Interna. https://www.mp.pl/interna/ [Accessed August 7, 2024]

4. Castelo-Branco L, Pellat A, Martins-Branco D, Valachis A, Derksen JWG, Suijkerbuijk KPM, Dafni U, Dellaporta T, Vogel A, Prelaj A, et al. ESMO Guidance for Reporting Oncology real-World evidence (GROW). Annals of Oncology (2023) 34:1097–1112. doi: 10.1016/J.ANNONC.2023.10.001/ATTACHMENT/1DE7E9A7-7499-484F-A53B-2901B1714FBA/MMC1.DOCX

5. Common Terminology Criteria for Adverse Events (CTCAE) v5.0. https://ctep.cancer.gov/protocoldevelopment/electronic_applications/docs/CTCAE_v5_Quick_Reference_5x7.pdf [Accessed January 21, 2025]
